# DECIPHER: harnessing local sequence context to improve protein multiple sequence alignment

**DOI:** 10.1186/s12859-015-0749-z

**Published:** 2015-10-06

**Authors:** Erik S. Wright

**Affiliations:** Department of Bacteriology, University of Wisconsin-Madison, Madison, WI 53715 USA; Wisconsin Institute for Discovery, University of Wisconsin-Madison, 330 N. Orchard St., Madison, WI 53715 USA

**Keywords:** Multiple sequence alignment, Secondary structure predictions, Large scale alignment, Benchmark datasets, Modeling gap penalties

## Abstract

**Background:**

Alignment of large and diverse sequence sets is a common task in biological investigations, yet there remains considerable room for improvement in alignment quality. Multiple sequence alignment programs tend to reach maximal accuracy when aligning only a few sequences, and then diminish steadily as more sequences are added. This drop in accuracy can be partly attributed to a build-up of error and ambiguity as more sequences are aligned. Most high-throughput sequence alignment algorithms do not use contextual information under the assumption that sites are independent. This study examines the extent to which local sequence context can be exploited to improve the quality of large multiple sequence alignments.

**Results:**

Two predictors based on local sequence context were assessed: (i) single sequence secondary structure predictions, and (ii) modulation of gap costs according to the surrounding residues. The results indicate that context-based predictors have appreciable information content that can be utilized to create more accurate alignments. Furthermore, local context becomes more informative as the number of sequences increases, enabling more accurate protein alignments of large empirical benchmarks. These discoveries became the basis for DECIPHER, a new context-aware program for sequence alignment, which outperformed other programs on large sequence sets.

**Conclusions:**

Predicting secondary structure based on local sequence context is an efficient means of breaking the independence assumption in alignment. Since secondary structure is more conserved than primary sequence, it can be leveraged to improve the alignment of distantly related proteins. Moreover, secondary structure predictions increase in accuracy as more sequences are used in the prediction. This enables the scalable generation of large sequence alignments that maintain high accuracy even on diverse sequence sets. The DECIPHER R package and source code are freely available for download at DECIPHER.cee.wisc.edu and from the Bioconductor repository.

**Electronic supplementary material:**

The online version of this article (doi:10.1186/s12859-015-0749-z) contains supplementary material, which is available to authorized users.

## Background

Multiple sequence alignment (MSA) is a ubiquitous task in biology, and has a wide variety of applications including homology detection [1], predicting residue couplings [2], finding evolutionarily important sites [3], oligonucleotide design [4], and phylogenetics. A multiple sequence alignment may reveal many aspects about a gene: which regions are constrained, which sites undergo positive selection [5], and potentially the structure of its gene product [6]. Many of these applications depend on the correct alignment of thousands of diverse sequences. A variety of methods have been developed to provide more accurate alignments [7–9], yet many of these approaches are not amenable to aligning thousands of sequences in a reasonable amount of time. Furthermore, performance tends to decrease dramatically beyond a certain point as more sequences are added to the input set [10]. Thus, the accurate alignment of large numbers of sequences remains an unsolved challenge that is frequently encountered in modern datasets.

It is generally believed that the poor scalability of alignment can be attributed to the build-up of error or the increasing level of ambiguity as more-and-more sequences are aligned. Two main strategies have been proposed to combat the loss in quality as alignments grow in size. The first strategy is to use a chained guide tree, which is efficient to construct and allows reasonable accuracy to be maintained on large empirical datasets (>1,000 sequences) [11]. However, this approach performs poorly on simulated sequence alignments [12], and may not be applicable for phylogenetic analyses [13]. The second strategy is to use an iterative divide-and-conquer approach that shows good performance on simulated sequence sets, but performs comparably to other methods on large empirical protein benchmarks [14]. A possible third strategy, proposed here, is to shift reliance onto structural information as alignments become larger. Since structure is more conserved than primary sequence, it is possible that structure-based alignment will maintain accuracy even as sequence-based alignment loses integrity.

MSA programs are typically optimized and assessed based on their ability to recreate the alignments in benchmark datasets. In this way, benchmarks determine the objective to which alignment programs strive to attain. There is an ongoing debate over whether simulated, structural, or other types of benchmark are preferable [15]. Simulated alignments are generated by “evolving” sequences along a predetermined tree under a model of substitution. Therefore, the complete evolutionary history of the sequences is known and the entire alignment can be used as a reference. In typical simulations, the choice of insertion and deletion rates across sites is specified, a substitution matrix is used, covariation between positions is ignored, and there is no selective pressure on the tertiary structure. Furthermore, real sequence sets often include spurious (e.g., chimeric [16]) sequences, sequencing errors, uneven taxon sampling, rearrangements, and uneven lengths that have largely been neglected in studies relying on simulations.

In contrast, many structural benchmarks have been built from related RNA or protein tertiary structures that have been superimposed to provide an empirical alignment that is free of many of the simplifications of simulated alignments. By this definition residues in the same column of an alignment should occupy the same structural position in space. A major downside of structural benchmarks is that “gappy” regions are typically not considered in scoring because they are not superimposable in space [17]. Some downstream applications of multiple sequence alignment may be especially sensitive to false homologies in gappy regions, such as tree building and the detection of positive selection [18, 19]. Nevertheless, structural benchmarks have generally been preferred over simulated benchmarks, resulting in an emphasis on the maximization of true homologies in “core blocks” (homologous regions), with less regard for false homologies.

The focus on maximizing true homologies has been furthered by a reliance on Q-score for performance comparisons with structural benchmarks. Q-score is defined as the average pairwise fraction of reference homologies that are also found in the test alignment (i.e, the alignment program’s output). Q-score does not directly penalize for aligning positions that are unaligned in the reference, also known as over-alignment [20]. Over-alignment can be quantified using the Modeler score (here termed M-Score), which is the fraction of aligned homologies that are also aligned in the reference [21]. A higher M-score indicates fewer false homologies, and vise-versa. The M-score does not penalize for under-alignment [20], as the correct alignment of only one position would result in a perfect M-score (i.e., 1). Hence, it is necessary to compare both true and false homologies when judging alignment performance.

Assessment of over-alignment is one step in the ongoing effort to create more biologically meaningful alignments [22]. Other efforts have focused on specific sequence features that may be present in some alignments but are neglected by most alignment programs. This has resulted in specialist alignment programs for different mutational events, such as long tandem repeats [23], domain rearrangements [24], and inversions [25]. Prevalent sequence features, such as short repeats and the local sequence context around insertions and deletions, have been identified as informative, yet are largely ignored by alignment programs [26]. In contrast, one source of information that has received significant attention is the use of secondary structure to provide a stronger biological basis for the alignment process. Those programs that have integrated secondary structure predictions into alignment have shown noteworthy gains in Q-score [27–31].

However, these gains have come at a cost because secondary structure is time consuming to accurately predict, which prevents these methods from scaling to a large number of sequences. Presently none of the alignment programs that use predicted secondary structure can align a thousand or more sequences in a reasonable amount of time [29]. This inefficiency is due to the need to find and align many sequences that are related to each sequence for which secondary structure is being predicted. Using the most accurate secondary structure predictions in sequence alignment therefore indirectly incorporates more sequence information into the alignment process. An alternative to this approach is to directly add more sequences to those being aligned, which has also been shown to substantially improve the accuracy of aligning small sequence sets [32]. Both of these approaches leverage large external databases of sequences that may not provide additional information when the input set is already large or all-encompassing.

In this study, I began by comparing the accuracy of structural benchmarks that would form the foundation for the rest of the study. Next, I investigated whether it was possible to efficiently integrate secondary structure predictions with negligible added time and no additional sequences other than those being aligned. To do this I relied on less accurate, but very fast, predictions made using the GOR method [33] for secondary structure prediction. The GOR method provides the probability of a residue being in helix (H), β-sheet (E), or coil (C) conformation based on local sequence context. Drawing inspiration from the GOR method, I created a model of gap placement that was also based on local sequence context. These features became the basis of a new program for multiple sequence alignment named DECIPHER. Finally, I compared DECIPHER’s performance with that of other popular alignment programs on high-quality structural benchmarks.

## Methods

### Secondary structure assignments

To compare empirical benchmarks, secondary structure assignments according to DSSP [34] were downloaded from Pfam [35] for proteins with solved structures. Pairs of sequences in each reference set were replaced with their corresponding secondary structure to generate an alignment of secondary structure states. A multiple alignment of *n* sequences therefore resulted in (*n*^2^ - *n*)/2 different pairwise alignments. The pairwise secondary structural identity of each of these alignments was calculated and used to compare benchmarks. Secondary structure identity was defined as the number of columns with matching secondary structure (8-state DSSP) normalized by the maximum number of matches possible. The large number of data points was simplified for plotting by finding the shortest contour line on the kernel density surrounding 75 % of points. The R programming language [36] was used for all analyses. *P*-values were calculated using the Wilcoxon signed rank test in R [36].

**Fig. 1:**
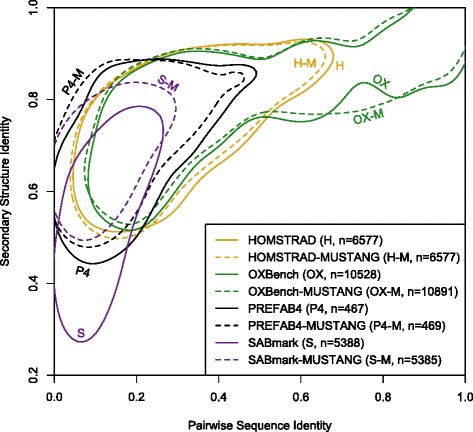
Comparison of structure-based benchmarks commonly used to rank sequence alignment programs. Each contour line surrounds the densest 75 % of points representing pairwise alignments in the benchmark. Structural identity is based on matching 8-state DSSP [34] secondary structure assignments (see Methods). Perfect secondary structure agreement would result in a score of 1 on the y-axis. Reference alignments exhibit decreased structural similarity as the distance between sequence pairs increases. Realignments using MUSTANG [40] showed improved quality in some cases, especially relative to the original SABmark [43] reference alignment

For secondary structure predictions, the GOR method was re-implemented as the DECIPHER function “PredictHEC”, and used automatically during alignment of amino acid sequences. The GOR method was trained on the dataset in Li et al. [37], which was reduced from 8-states to 3-states according to the convention: H = (G, H, I), E = E, C = other [34]. In the GOR method, probabilities at a site are assigned to each of the three states while taking into account a window of seven residues to either side of the site. Contributions from single residues and pairs of residues were considered, as in version IV of the GOR algorithm [33]. Probabilities were normalized relative to the background distribution in accordance with version V of the algorithm [38], which results in a modest improvement over version IV predictions. Only unaligned single sequences were used in the calculation of 3-state probabilities that were used in sequence alignment.

### Multiple sequence alignment benchmarks

HOMSTRAD [39] multiple alignments were downloaded on February 20^th^, 2015 from the website mizuguchilab.org/homstrad. The HOMSTRAD alignments were realigned using MUSTANG (v3.2.1) [40]. All other benchmarks were downloaded as part of the bench (v1.0) collection from www.drive5.com/bench. This collection includes OXBench [41], PREFAB (v4.0) [42], and transitively-consistent alignments from SABmark (v1.65) [43] in both their original form and realigned with MUSTANG [40]. These benchmarks were compared (see Results), and PREFAB and HOMSTRAD were selected for benchmarking MSA programs due to their high quality and breadth of sequence identities. The selected benchmarks required slight modification before they could be used to assess the alignment of large numbers of sequences.

To create HOMSTRAD-mod, columns of the alignment that were in agreement between the original and MUSTANG alignments were kept uppercase to define core blocks. Therefore, HOMSTAD-mod alignments are identical to those of HOMSTRAD in the regions used in scoring. Alignments with (i) less than 25 % of their length in core blocks, (ii) a total width of less than 30 sites, or (iii) having greater than 80 % average pairwise identity were removed. Benchmarks were supplemented with full-length Pfam [35] sequences downloaded from each set’s corresponding Pfam family. The matching Pfam homologous region was required to be less than three times the width of the respective reference sequences. Reference sets with fewer than 100 supplemental sequences were removed. PREFAB-mod reference pairs were left untouched from the original PREFAB sequences realigned with MUSTANG. The final benchmarks contained 717 and 399 reference sets in HOMSTRAD-mod and PREFAB-mod, respectively. All benchmarks created for this study are available from DECIPHER.cee.wisc.edu/Download.html.

When comparing performance, input reference sets were generated by randomly selecting a predefined number of supplemental sequences from the pool of available Pfam sequences. These supplemental sequences were added to the reference sequences to reach the intended total number of sequences in each input set (between 125 and 4,000). After alignment, the supplemental sequences were removed and the remaining (reference) sequences were tested for alignment accuracy. Only one randomly selected set of supplemental sequences was used per alignment size, up to the maximum number of sequences available for each set. The smallest sets of 2 sequences were created by randomly selecting a pair of sequences from each reference set. All alignments were scored using qscore [42] with optional parameters “-ignoretestcase -cline -modeler”. These parameters specify that only uppercase letters (core blocks) in the reference alignment are used in scoring, and that qscore should output the Cline shift-score [44] and Modeler score (M-score) [21].

### Gap databases

Sequence pairs in the One Gap Database [26] were translated and realigned with the objective of creating a high accuracy unbiased set of aligned sequences with gaps. The realignment procedure, described as follows, did not include a model of gap placement. First groups of sequences were used to create a multiple alignment. The most similar pairs of sequences with different internal gap patterns were then realigned to remove any artifacts from the multiple alignment. Pairs with gaps remaining after pairwise alignment were kept, and their gaps were marked to prohibit the reuse of gaps in the same position in other pairs. This process was repeated for each protein family to generate a large set of pairwise alignments with different internal gaps.

To prevent incorrect gap placements, sequence pairs were required to contain gap events separated by at least 20 residues, and have greater than 50 % sequence identity. To mitigate the effect of ambiguous gap placements in repetitive regions, the sequence pairs were realigned in reverse orientation and then reversed again to generate a complementary alignment. Finally, local alignments that were equivalent (e.g., AA/A- and AA/-A) were expanded into all possible permutations and weighted to split the permutations evenly. The same process was repeated with Pfam families to generate a complementary set of high quality gap placements. The final sets contained 58,509 gaps from the One Gap Database, and 46,168 from the Pfam database. Observed residue frequencies were converted into log-odds in third-bits based on the formula: log(observed probability/background probability)*3/log(2). Log-odds scores were highly correlated between the two datasets (R^2^ = 0.88), so the average score was used for model parameters.

### Alignment programs

DECIPHER is an R [36] package with functions for primer design [45], probe design [46], and other bioinformatics tasks. In this study the DECIPHER software was extended to include multiple sequence alignment with the function “AlignSeqs”, which can align a set of DNA, RNA, or amino acid sequences. DECIPHER also includes functions for alignment of DNA sequences via their translation (“AlignTranslation”), and the merging of two existing alignments (“AlignProfiles”). See the Additional file 1 text for a complete description of the DECIPHER algorithm. DECIPHER was written in the C and R programming languages, and is available from DECIPHER.cee.wisc.edu or BioConductor [47].

The following programs were compared in this study:

- Clustal Omega (v1.2.0) [48]
- DECIPHER (v1.14.4)
- MAFFT (v7.22.0) [49]
- MUSCLE (v3.8.31) [50]
- PASTA (v2.2.7) [14]
- PROMALS [27]

Default parameters were used for all programs with the exception of MUSCLE and PASTA, which required changing the maximum number of iterations. For MUSCLE, “maxiters” was change from 16 to 2 for sets of 500 or more sequences as recommended by the developers. For PASTA, the parameter “iter-limit” was changed to 1 for sets of 500 or more sequences. Attempts to use the default value of 3 proved prohibitively time consuming on larger sets. For MAFFT the “auto” option was used to automatically switch between different progressive and iterative strategies based on the number and length of input sequences. Timings for all sets were determined on a 2.2 GHz Intel Core i7 with 8 GB of RAM using a single processor. For consistent timing comparisons, PASTA and PROMALS were configured to use only one processor.

## Results

### Choosing high quality reference alignments for benchmarking

Different benchmarks often result in contrasting optimal parameters (e.g., gap opening and extension penalties) and an incompatible performance ranking of alignment programs [51]. For these reasons, the choice of benchmark is of utmost importance when developing and comparing algorithms for sequence alignment. To choose alignment benchmarks for this study, I began by comparing secondary structure concordance across common benchmarks. This method of comparison requires that the secondary structure of reference sequences be available, which excludes the popular BAliBASE benchmarks [52] because the corresponding secondary structure of most BAliBASE sequences is unknown [53]. Although secondary structure agreement alone is insufficient to ensure a high quality benchmark, a lack of agreement can be an indication of alignment inaccuracy.

It is expected that better reference alignments will have a greater percentage of aligned residues with identical secondary structure. However, some disagreement in secondary structure is anticipated due to both intrinsic difficulties in assigning secondary structure [54] and challenges inherent to aligning distantly related tertiary structures [21, 55]. Figure 1 shows the fraction of secondary structure agreement versus pairwise sequence identity for four common amino acid benchmarks. The SABmark [43] and PREFAB [42] benchmarks contain the greatest fraction of their sequences in or below the “twilight zone” of 20 to 35 % sequence identity, while the emphasis of OXBench [41] is on less challenging alignments. PREFAB appears to be significantly better aligned overall than SABmark, despite both references covering a similar range of sequence identities. For sequences with less than 10 % identity, PREFAB has 13.4 % greater structural identity (*p* < 1e-15) than SABmark. These findings are in agreement with a previous study [53] that found PREFAB to be the best benchmark designed specifically for comparing MSA programs, although PREFAB is known to contain errors [56].

All columns of the alignments were used to assess the overall accuracy of each benchmark rather than only using core blocks (homologous regions), which are typically delineated by uppercase letters. The choice to use the entire alignment was made because: (i) the definition of core blocks varies between benchmarks, (ii) some scoring procedures make use of the entire alignment [44], (iii) pairwise distance is calculated using the whole alignment, and (iv) the HOMSTRAD [39] and SABmark benchmarks do not delineate core blocks. Core blocks in PREFAB were assigned based on the agreement between two different structural alignment programs. This motivated me to look at the difference between the original benchmarks and the same sequences realigned with the sequence-independent structural alignment program MUSTANG [40]. Realignments with MUSTANG exhibited greater secondary structural congruence than the original benchmarks, except in the case of HOMSTRAD (Fig. 1). In particular, SABmark had 11.2 % higher secondary structure identity after realignment with MUSTANG (*p* < 1e-15). This result supports the use of the HOMSTRAD database as an alignment benchmark even though it was not originally intended for this purpose.

Since the number of sequences with known structure is small relative to the number of available sequences, most benchmarks are supplemented with additional unaligned sequences that are not considered in scoring. PREFAB reference alignments are supplemented with additional sequences found using PSI-BLAST searches [57] with the reference sequences. HOMSTAD sequences are commonly supplemented with other sequences belonging to the same Pfam [35] family [11, 48]. I compared these two approaches by randomly selecting sequences from the Pfam family corresponding to the PREFAB reference sequences. After generating an alignment with the same number of supplementary sequences, a neighbor joining tree was constructed to determine the breadth of the added sequences. The average tree length was 1.6 times longer for random Pfam sequences than those included with PREFAB (*p* < 1e-15). This indicated that extending the input set in a way that is not directly dependent on the reference sequences results in the greatest diversity of supplemental sequences.

It is unclear which reference benchmark most adequately reflects a typical user’s sequences, and the wide diversity of MSA applications probably spans most of the alignment scenarios found in benchmarks. SABmark sets cover a narrow range of sequence identities, while OXBench focuses on closely related sequences that are easier to align. Due to both alignment quality and breadth of sequence identities, I chose to continue the rest of this study with slightly modified versions of the original PREFAB and HOMSTRAD datasets, called PREFAB-mod and HOMSTRAD-mod (see Methods). To supplement the modified benchmarks, I added full-length sequences belonging to the same Pfam family. Full-length sequences were used rather than only the shared domain to make the alignments more challenging and to represent a greater variety of potential usage scenarios. Oftentimes sequences being aligned have varying lengths because they cover overlapping regions of a gene, or were trimmed differently based on their quality scores at each terminus.

### Scalable incorporation of secondary structure into alignment

Despite the close connection between secondary structure and sequence alignment, most popular protein alignment programs do not predict structural information. The main drawback of secondary structure prediction is that it is slow to accurately compute, which prevents it from scaling to the alignment of hundreds of sequences in a reasonable amount of time [29]. Less accurate secondary structure predictions can be obtained very rapidly using single-sequence approaches that do not rely on constructing a multiple alignment with homologous sequences. The GOR method is one of the most accurate given a single sequence [38]. In this method secondary structure is assigned to one of three states: helix (H), sheet (E), or coil (C) based on the local sequence context surrounding a residue. This approach has the advantage that it is extremely fast (< 1 % of the time required for alignment), provides a probability value for each state, and offers about 65 % accuracy [33].

To integrate secondary structure predictions into the dynamic programming framework for profile-profile alignment, I added a new 3 × 3 symmetric matrix representing the log-odds of aligning an H, E, or C in one sequence with another position assigned to H, E, or C in a second sequence. Coupling this matrix with the probability assigned to each of the three structural states allowed for profile-profile alignment of the secondary structures. The score obtained from aligning secondary structure profiles augmented the traditional substitution matrix based score determined from the primary sequences (see Additional file 1 text). In this way, primary and secondary structure agreement can be maximized simultaneously.

**Fig. 2:**
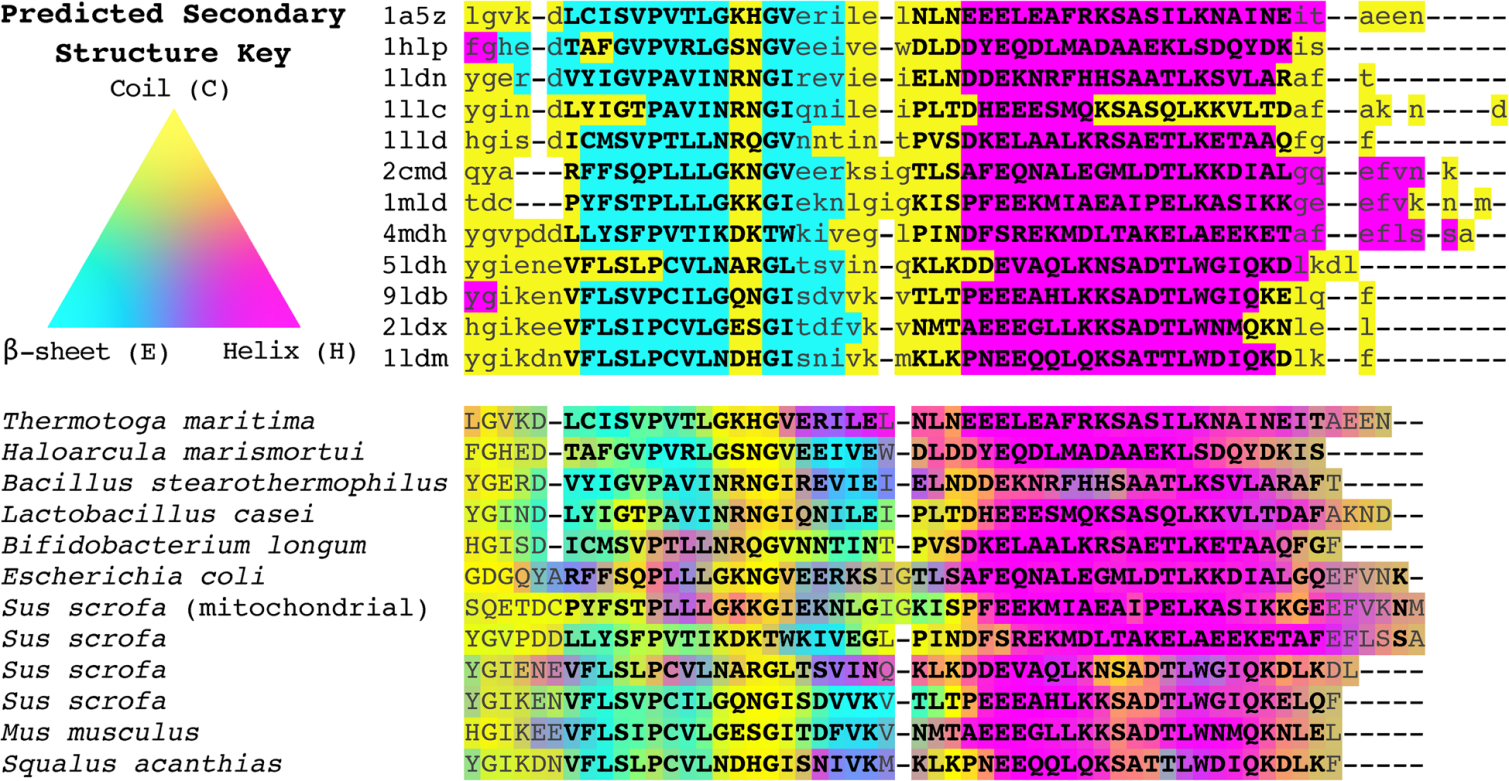
C-terminal end of alignments of the lactate/malate dehydrogenase protein family (Pfam [35] accession no. PF00056) colored by predicted secondary structure. The top alignment (sequences named by PDB ID) is from the HOMSTRAD-mod benchmark colored by DSSP assignments [34], with upper-case letters denoting core blocks. The lower alignment shows the same sequences (named by organism) realigned with DECIPHER and colored according to 3-state probabilities predicted by the GOR method [33]. Columns of the lower alignment in bold exactly match columns of the upper reference alignment

Figure 2 displays an example alignment of the lactate/malate dehydrogenase protein family (Pfam [35] accession no. PF00056; HOMSTRAD “ldh” family) obtained using this approach. The DSSP [34] assignments are in general agreement across the HOMSTRAD-mod alignment, which is based on the known tertiary structures of these proteins. Predictions made with the GOR method reflect these secondary structure assignments with some discrepancies. The GOR predictions guide the DECIPHER alignment, which exactly matches the reference alignment in regions defined as core blocks, denoted by uppercase letters in the upper alignment of Fig. 2. Regions of the reference alignment that fall outside of core blocks are not used in determining accuracy and differ from the DECIPHER output in some columns.

**Fig. 3:**
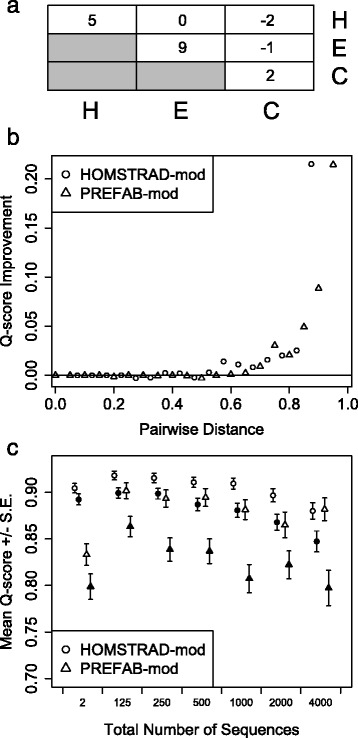
**a** Optimized structure matrix for pairings between helix (H), β-sheet (E), or coil (C) states. **b** Repeated values are grayed-out since the matrix is symmetric. After incorporating this matrix into alignment, the average improvement in Q-score on pairwise alignments was greater for distant pairs. **c** Alignments using the structure matrix (open symbols) showed little decline in accuracy as the number of input sequences increased relative to alignments made without structural predictions (closed symbols). Across all alignment sizes, the use of secondary structure improved Q-score (*p* < 1e-5 for all). Similarly, the improvement in Q-score (separation between open and closed symbols) increased as more sequences were aligned

One advantage of using a small 3 × 3 secondary structure matrix is that the number of free parameters is far outnumbered by the number of informative data points, which makes estimation error negligible (Additional file 1: Figure S1). To find optimal values for each of the 6 distinct parameters in the matrix, I performed a grid-search for the solution that resulted in the best-scoring alignments based on the sum of Q-score and M-score on a subset of HOMSTRAD-mod consisting of 238 reference sets. At the optimum between over-alignment and under-alignment, any gain in Q-score is outweighed by the corresponding loss in M-score, and vise-versa. The optimized secondary structure matrix is shown in Fig. 3a. E-states are very likely to be aligned, as reflected in the large contribution of E/E pairings to the secondary structure score. The GOR method tends to under-predict β-sheets, resulting in a low fraction of E-states in most sequences [33].

Next, I asked whether incorporation of secondary structure improved sequence alignment, and how this scaled with the number of sequences being aligned. Averaged across all sizes of sequence sets, incorporation of secondary structure resulted in a 5.3 % improvement in Q-score on PREFAB-mod and 2.1 % on HOMSTRAD-mod. This substantial increase in Q-score came at the expense of a 0.4 % decrease in M-score on PREFAB-mod and a 0.3 % decrease on HOMSTRAD-mod. Therefore, the fraction of homologies that are correctly aligned slightly decreased, while the total number of correctly aligned homologies substantially increased. Unsurprisingly, the largest gains were on divergent reference sets where there is the most room for improvement, and essentially no gain was made on references with less than 60 % average distance between pairs (Fig. 3b). Secondary structure predictions provided a greater benefit on PREFAB-mod because a larger fraction of its reference sequences are over 60 % distant.

Interestingly, the improvement from incorporating secondary structure increased as more sequences were aligned (Fig. 3c). On the smallest sets of 2 sequences there was a 3.4 % improvement on PREFAB-mod and 1.2 % on HOMSTRAD-mod. On large 4,000 sequence sets the advantage increased to 8.5 % and 3.3 %, respectively. Therefore, incorporating secondary structure partially counteracted the decrease in score that is typically observed with larger alignments [10]. This behavior mirrored that of secondary structure prediction, where accuracy increases as more sequences are used in the calculation [38]. For this reason, the most accurate secondary structure prediction algorithms make use of multiple alignments. Similarly, here the initial secondary structure predictions lack accuracy since they are obtained from single sequences. As more sequences are aligned, these probabilities are averaged to increase their accuracy and better guide the alignment. This is in contrast to primary sequence, where additional sequences inevitably result in more ambiguity, which in part causes a loss of signal that manifest in poor quality alignment of ambiguous profiles.

### Including a model of indel probability to improve gap positioning

Motivated by the improvement obtained from incorporating local sequence context via secondary structure predictions, I next asked whether the same approach could be applied to gap placement. Previous research has revealed that insertions and deletions (indels) are more likely to occur adjacent to certain amino acids [26] and in exposed coil regions [58]. For this reason it is common to decrease the cost of opening a gap in hydrophilic stretches [59], or alternatively to increase the cost in hydrophobic regions [50] that are likely to be buried in the protein’s constrained core. To my knowledge, a more sophisticated model of gap likelihood based on local context has not been applied to sequence alignment. To this end I used the One Gap Database [26] to calculate the relative frequency of indel events based on the residues to the left and right of a central gap. This frequency information was then converted into log-odds scores according to the background frequency of each amino acid.

**Fig. 4:**
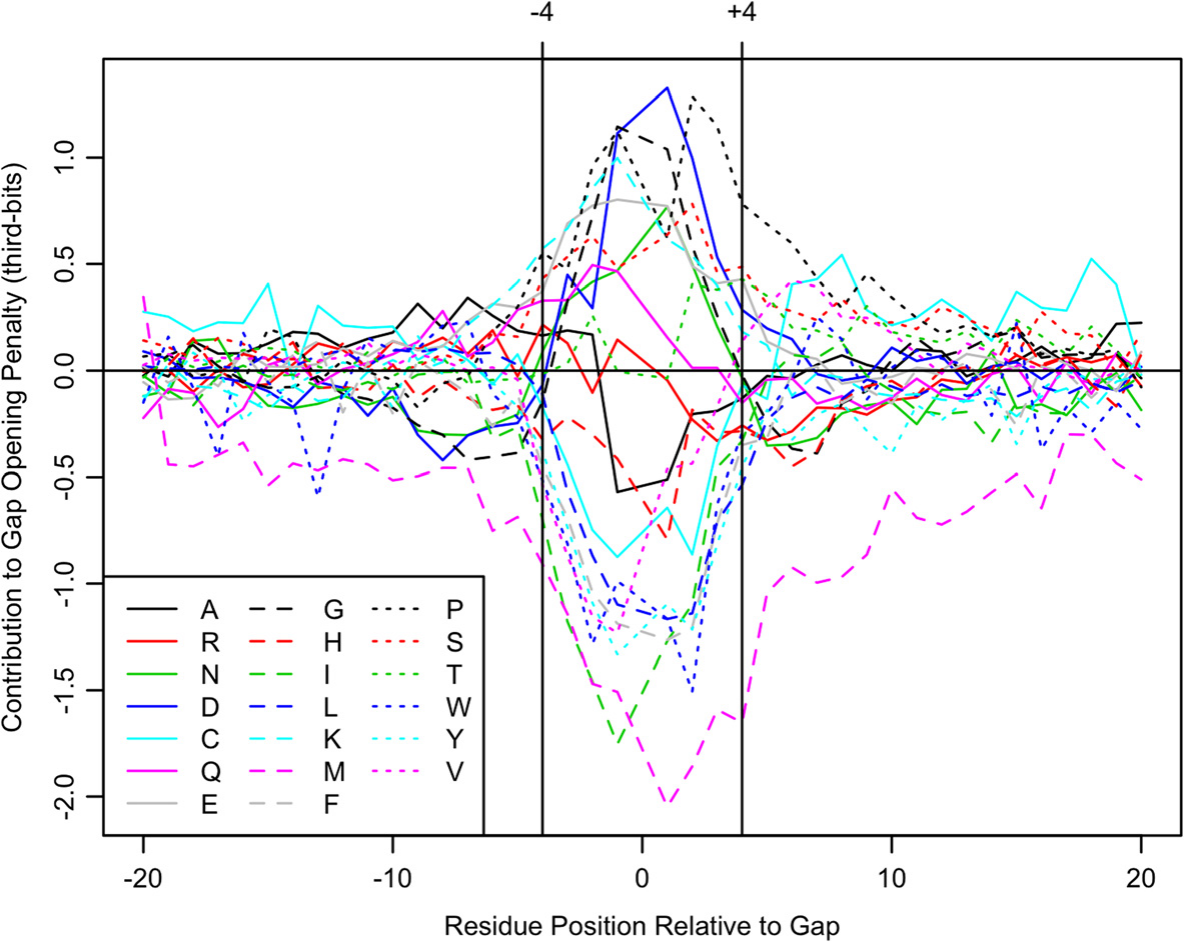
Contribution of local sequence context to the cost of opening a gap in the alignment. Hydrophobic residues greatly decrease the likelihood of a gap, whereas hydrophilic and “structure-breaking” residues increase the likelihood of a gap. In the gap model, positions located within four residues were used to modulate the cost of opening a gap at position zero

Figure 4 shows the contribution of nearby amino acids to the likelihood of a gap at position zero. As expected, hydrophobic residues (FMILYW) greatly decrease the likelihood of a gap. Hydrophilic and “structure-breaking” (e.g., P) residues increase the chance of an adjacent gap, albeit with less of an effect than hydrophobic residues. Since the log-odds scores are in the same units as the substitution matrix (third-bits), they can be directly applied to modulate gap-opening and gap-closing costs at any position based on its local sequence context (Additional file 1: Table S1). I evaluated different window sizes for including this information, and found that the best window stretched from position -4 to +4 relative to the central gap. Hence, the cost of creating a gap at any position is the original gap cost plus a score that is modulated based on the residues to either side of the gap (see Additional file 1).

Next, I calculated log-odds scores for the residues opposing the gap (in the ungapped sequence), and found that these positions displayed a small bias in amino acid content (Additional file 1: Table S1). There was a moderate correlation between the log-odds scores for positions to the left or right of the gap and the residues opposing the gap (R^2^ of 0.69 and 0.64, respectively). However, in this case there was no apparent difference between locations within the gapped region. For this reason I chose to simply modulate the gap extension cost based on the average scores for the “gapped” residues in a position-independent manner. Altogether, this probabilistic model of opening and extending a gap adjusts the gap penalty within a range of about +/- 20 % at each position.

To expand this model of gap placement based on local sequence context, I next investigated the effect of short sequence patterns. Repeats are a major source of length variation in biological sequences [60] and are commonly found across all branches of life [61]. Repeats have a wide variety of forms, including short microsatellite repeats of a single codon and longer tandem repeats of regions that may evolve through mutation to become mismatched over time [62]. Longer repeats can be aligned with specialized programs [23] that employ tandem repeat finding algorithms [63]. Short patterns are typically neglected as insignificant by these programs due to their frequent occurrence in sequences. However, Chang and Benner [26] found that short dipeptide repeats (e.g., AA) were more common than expected around gaps, potentially offering a means of modulating gap costs. To investigate this effect, I examined the occurrence of different sequence patterns in the One Gap Database.

Dipeptide repeats (runs of 2 identical amino acids) surrounding gaps were only slightly more likely (< 1 third-bit) than expected by chance. However, gaps were substantially more likely to occur around runs of three or longer (e.g., AAA), as shown in Additional file 1: Figure S2. This effect was particularly pronounced in the sequence without the gap, indicating that gaps are often present because one sequence has a longer run than another. Surprisingly, gaps were less likely to occur at the position after the start of a run in the opposing sequence (e.g., AA/A-), regardless of the run’s length. Although the mechanism for this occurrence is unknown, it may be due to a biological role for dipeptide repeats that results in their conservation. A similar investigation of heteropeptide repeats with periodicity 2 (e.g., ACAC) to 6 did not reveal a strong bias towards gaps (Additional file 1: Figure S2). Therefore, I chose to extend the gap model to modulate the gap opening cost at positions before and immediately after the start of a run in the opposing sequence.

Overall, employing this model of gap placement resulted in a modest improvement of 0.5 % on PREFAB-mod (p < 1e-4) and 0.3 % on HOMSTRAD-mod (p < 1e-3). The improvements in Q-score were matched by 0.2 % increases in M-score on both benchmarks. These changes in score were unexpected, as structural benchmarks do not consider most gapped regions since they often occur in parts of the structure that are difficult to superimpose [17], and repeats tend to be found in disordered protein regions [64]. Although, evolutionary simulations offer a means of scoring gapped regions, such simulations currently do not include a context dependent model of gap likelihood. Therefore, it is possible that the placement of gaps improved more than reflected by the modest increase in scores, but there currently exist no adequate way of measuring the actual advantage of incorporating a sophisticated gap model into alignment.

### Comparison of DECIPHER to other programs for MSA

**Fig. 5:**
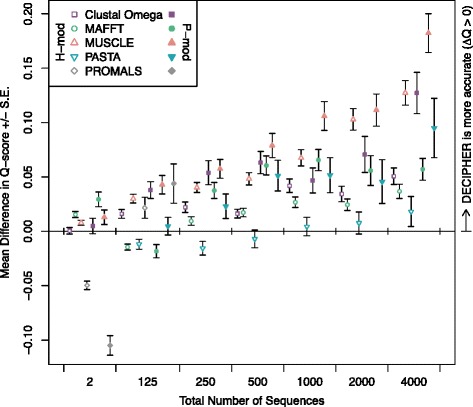
Performance of popular multiple sequence alignment programs relative to DECIPHER on the HOMSTRAD-mod (H-mod) and PREFAB-mod (P-mod) benchmarks. PROMALS [27] exhibited the best performance on the smallest sets of two sequences. MAFFT [49] had the best performance on small input sets of 125 sequences, where it uses a much slower consistency-based strategy. MUSCLE [50] showed the worst performance on larger sequence sets. DECIPHER’s performance relative to other programs improved as more sequences were aligned

Having successfully integrated context-awareness into the DECIPHER software for sequence alignment, I next compared its performance to other state-of-the-art alignment programs. First, I chose to benchmark DECIPHER against three popular programs capable of efficiently aligning thousands of sequences: Clustal Omega [48], MAFFT [49], and MUSCLE [50]. These programs are regularly employed in a variety of different studies, and have become the de facto standard for comparison on benchmarks. Figure 5 shows the performance of each program relative to DECIPHER for increasing numbers of input sequences. The performance ranking is in strong agreement between the HOMSTRAD-mod and PREFAB-mod benchmarks, yet there is a greater spread between programs on PREFAB-mod because it contains a larger fraction of sequences in or below the twilight zone.

When only two sequences were aligned from each benchmark, the alignment programs all gave similar results, with MAFFT showing the lowest accuracy. In the sets of 125 sequences, DECIPHER is ranked second behind MAFFT. For input sets of this size, MAFFT uses its most accurate consistency-based algorithm (L-INS-i) that is not scalable to larger sequences sets. Beyond 125 input sequences, DECIPHER clearly outperforms the other three programs (Additional file 1: Table S2), and its lead improves as more sequences are aligned (Fig. 5). This reflects the fact that DECIPHER’s accuracy stays relatively constant with increasing numbers of sequences (Fig. 3), which is partly attributable to its use of secondary structure during alignment. Clustal Omega, MAFFT, and DECIPHER all have similar M-scores across the range of input sizes (Additional file 1: Figure S3). MUSCLE had the poorest performance, with substantially worse Q- and M-scores for all but the smallest input sequence sets. Furthermore, although Q-score, total column score (TC-score), and Cline shift-score [44] sometimes give conflicting performance rankings, these three statistics strongly agreed on both benchmarks (Additional file 1: Figures S4 and S5).

Over-training to a single reference set has been a concern for some alignment programs [51], although both reference sets used here showed similar results. However, other programs may be better trained on the original benchmarks that are not based on the outputs of the MUSTANG structural alignment program. To verify that DECIPHER was not over-trained to MUSTANG’s outputs, I repeated the analysis using the original PREFAB reference pairs, which were aligned independently of MUSTANG. The unmodified PREFAB reference sequences showed strong secondary structure concordance, and therefore provide a high-quality alternative benchmark. Nevertheless, the results (Additional file 1: Figure S6) were very similar for both sets of reference sequences, indicating that DECIPHER’s performance was not closely tied to MUSTANG’s outputs.

I next compared DECIPHER to PASTA [14], which is a program intended to extend the accuracy of less-scalable algorithms to large alignments. PASTA works by dividing an alignment up into overlapping sub-problems that are each aligned with an accurate strategy, by default MAFFT’s L-INS-i consistency-based approach. These sub-alignments are merged using transitivity, and the process is repeated starting from a new guide tree. Interestingly, PASTA outperformed DECIPHER on sets of 125 and 250 sequences on HOMSTRAD-mod (Fig. 5), but was statistically indistinguishable on larger sets (Additional file 1: Table S2). However, DECIPHER substantially outperformed PASTA on PREFAB-mod, and its lead increased as more sequences were aligned. Furthermore, PASTA showed a large drop in accuracy with increasing alignment size. Table 1 shows that DECIPHER’s performance diminished the least of all alignment programs as alignment size increased.

**Table 1:** Change in average Q-score according to the number of sequences being aligned

	Total sequences		Clustal Omega	DECIPHER	MAFFT	MUSCLE	PASTA^a^
HOMSTRAD-mod^b^	Maximum score		0.898	0.910	0.920	0.881	0.921
	Change from max	2	−0.013	−0.02	−0.045	0.000	N/A
		125	0.000	0.000	0.000	−0.007	0.000
		250	−0.009	−0.011	−0.028	−0.028	−0.003
		500	−0.013	−0.014	−0.037	−0.028	−0.016
		1000	−0.035	−0.011	−0.049	−0.048	−0.026
		2000	−0.035	−0.019	−0.054	−0.088	−0.028
		4000	−0.069	−0.030	−0.077	−0.128	−0.049
PREFAB-mod^b^	Maximum score		0.875	0.908	0.923	0.855	0.900
	Change from max	2	−0.067	−0.085	−0.123	−0.047	N/A
		125	0.000	0.000	0.000	0.000	0.000
		250	−0.022	−0.007	−0.057	−0.011	−0.015
		500	−0.022	−0.004	−0.068	−0.026	−0.029
		1000	−0.034	−0.023	−0.090	−0.056	−0.045
		2000	−0.069	−0.037	−0.107	−0.093	−0.080
		4000	−0.121	−0.026	−0.098	−0.156	−0.116

^a^Scores for aligning two sequences are listed as “N/A” because PASTA cannot perform pairwise alignment

^b^Results for the subset of reference alignments with at least 4,000 reference sequences are shown (297 alignments for HOMSTRAD-mod and 201 alignments for PREFAB-mod)

**Fig. 6:**
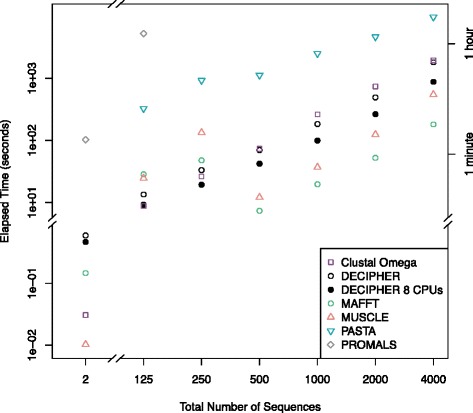
Average execution time according to the number of sequences being aligned (note the axis breaks and log-scale). PROMALS [27] was substantially slower than the other programs that do not rely on a large external database of sequences. MAFFT [49] was the fastest program for large sequence sets. PASTA was the slowest program tested for aligning large sequence sets, requiring an average of 2.7 h to align 4,000 sequences. A noteworthy speed improvement was obtained with DECIPHER by using multiple processors

Finally, I compared DECIPHER’s performance to PROMALS [27], which is a program that relies on more accurate secondary structure predictions obtained from PSIPRED [65]. PROMALS first performs PSI-BLAST searches with representative sequences from the input set, and then uses accurate secondary structure predictions with a consistency-based approach to align the sequences. PROMALS greatly out-scored all of the other alignment programs on the smallest sets of two sequences, but its advantage disappeared once other sequences were added to the input set (Fig. 5). Furthermore, it was several orders of magnitude slower that the other aligners (Fig. 6), and testing input sets larger than 125 sequences proved prohibitively time consuming. More recent approaches that make use of solved protein structures are available, such as PROMALS3D [66]. However, it is unclear how to test such approaches on structural benchmarks, because the reference sequences are likely present in the same structure databases used by these programs.

DECIPHER was neither the slowest nor fastest program benchmarked for aligning each of the sequence sets (Fig. 6). MAFFT was generally the fastest program, except for the smallest sequence sets where it uses slower, more accurate strategies for alignment. The change in elapsed time is dramatic for MAFFT and MUSCLE beyond 250 sequences where more efficient strategies were used. PASTA was the slowest program, and required an average of 2.7 h to align 4,000 sequences. Both Clustal Omega and DECIPHER were able to align 4,000 sequences in about half an hour on average. Since guide tree computation is the limiting factor for large sequence sets, parallelization may be useful in such circumstances. For example, DECIPHER was about twice as fast when 8 processors were used (Fig. 6). DECIPHER’s maximal memory use was 2GB when aligning 4,000 sequences.

## Discussion

The accurate alignment of very large numbers of sequences has been a long-standing goal for sequence alignment programs. DECIPHER exhibited excellent performance in the range of hundreds to thousands of sequences, with little decrease from maximal accuracy. This number of input sequences is common in current investigations harnessing next generation sequencing or large online sequence repositories. Even greater numbers of sequences are often available, but the scalability of these techniques to ultra-large alignments was not assessed in this study for two reasons. First, extremely large sequence sets can likely be reduced to a more manageable size through the clustering of highly similar sequences into groups represented by consensus sequences. Second, it is questionable whether there currently exists a reasonable empirical benchmark for ultra-large alignments (> 10,000 sequences). The popular strategy employed here, of extending structural benchmarks with supplemental sequences, suffers from a dilution problem as the number of supplementary sequences begins to greatly outnumber the reference sequences.

It has been previously established that the vast majority of information indicating whether to align two positions is contained directly in the amino acid pairing. This has led to the assumption of positional independence that is the primary means for efficient alignment algorithms [67]. However, the results of this study show that local sequence context can be efficiently harnessed to further improve alignments. GOR secondary structure predictions are based solely on local residues, and are therefore an indirect means of incorporating contextual information. Previous direct attempts to break the independence assumption have been based on substitution matrices with quadruplets of amino acids [67]. However, direct approaches have failed to show an improvement in alignment quality, possible due to the extremely large number of parameters required to estimate the substitution matrix of all possible dipeptides (80,200 distinct values). Very large datasets such as BLOCKS [68] are still insufficient to accurately determine the frequency of many amino acid quadruplets [67].

My own attempts to construct a substitution matrix based on amino acid triplets also showed signs of estimation inaccuracy. However, testing this matrix did reveal a small improvement in Q-score, albeit far less than that of using secondary structure predictions. The GOR (version IV) method employed here uses two matrices of parameters, one based on single residues and the other on pairs of residues, which can be accurately estimated due to their relatively small size. Furthermore, reduction to a three-letter (H/E/C) alphabet that reflects an important property of the alignment enables local sequence context to be efficiently harnessed, because the contextual information only needs to be computed once per site and can then be reused under the dynamic programming approach to alignment. In contrast, using large substitution matrices requires re-computing the covariation score at every site, which is very inefficient and is not suitable for large sequence sets [67].

## Conclusions

The main finding of this study is that fast secondary structure predictions can be employed in a scalable manner to counteract the drop-off in accuracy associated with aligning more sequences. This effect can be explained by the fact that structure is more conserved than sequence and therefore remains a reliable predictor even as sequences diverge greatly. Secondary structure prediction algorithms exhibit a similar increase in accuracy as more sequences are used in the prediction. For example, accuracy of the GOR algorithm increases by 6 % when multiple sequences are used for prediction [38]. The same logic was applied in this study, as profiles of secondary structure predictions are progressively merged while sequences are aligned along the guide tree, resulting in improved group-level predictions that assist alignment. At the top of the guide tree, where the sequence profiles being merged are highly divergent, the secondary structure probabilities are more accurate because they are based on the entire group’s consensus prediction.

There is an inherent trade-off between true and false homologies, and the results of this study advocate for the comparison of both in the development and benchmarking of alignment algorithms. While it is common to report Q-score and TC-score, these two statistics are strongly correlated. In contrast, Q-score and M-score are not linearly related, and beyond a certain optimum one must be lowered to raise the other. Analyses of alignment performance have often focused solely on quantifying true positives (i.e., Q-score), which has the potential to paint an unbalanced picture of alignment performance. Similarly, the choice of alignment benchmark was carefully analyzed in this study. The results showed that not all reference sets are equally well aligned, and therefore benchmarks should be compared in addition to alignment programs [53]. Treating all benchmarks as intrinsically equivalent risks developing algorithms that are trained for the wrong goal.

## Additional files

Additional file 1:
**This supplementary file contains a description of the DECIPHER algorithm, Figures** **S1–S6**, **and Tables** **S1–S2.** (PDF 719 kb)
