# Supplementary material for: DECIPHER: harnessing local sequence context to improve protein multiple sequence alignment
Source: BMC Bioinformatics. 2015 Oct 6;16:322. doi: 10.1186/s12859-015-0749-z (PMC4595117; doi:10.1186/s12859-015-0749-z)
Supplement: Additional file 1: — This supplementary file contains a description of the DECIPHER algorithm, Figures S1–S6, and Tables S1–S2. (PDF 719 kb) [file 12859_2015_749_MOESM1_ESM.pdf]

SUPPLEMENTAL INFORMATION

Description of the DECIPHER algorithm

The method can be separated into four stages:

1. INITIALIZATION:
   Prediction of secondary structure probabilities for all input sequences.
2. PRELIMINARY:
   Calculation of a crude guide tree based on shared k-mers, followed by progressive alignment.
3. ITERATION(S):
   Calculation of a UPGMA guide tree based on pairwise distances, followed by progressive alignment.
4. REFINEMENT(S):
   Repeated splitting of the alignment into two groups that are realigned. The highest scoring of the two alignments is kept.

In step 1, the probability that each residue is in helix (H), β-sheet (E), or coil (C) conformation is predicted for the input set of unaligned sequences. Three-state probabilities are calculated according to the GOR (version IV) algorithm for secondary structure prediction [1]. This method was re-implemented with minor modifications in the DECIPHER software, and made accessible via the "PredictHEC" function. The GOR method uses two matrices of parameters, which were calculated from the training dataset provided in the R package bamboo [2]. Testing of this method verified that it offers about 65% accuracy at 3-state prediction (H/E/C) based on single sequences as described by its authors [3]. Furthermore, predictions with the GOR method do not decrease in accuracy in leave-one-out cross validation, indicating that the parameters are converged. The normalized probability associated with each state was used during sequence alignment rather than the state prediction (i.e., H, E, or C).

In step 2, a guide tree is calculated based on shared k-mers. This approach is common to many algorithms for sequence alignment [4, 5]. The main difference in the DECIPHER algorithm is that the fraction of shared k-mers is calculated in accordance with the ordering of the sequences. In this way, k-mers must occur in the same sequence order rather than simply being present in both sequences. The advantage of using ordering is that it performs well for long sequences that are expected to share a large number of k-mers by chance. However, the ordered-approach is slower than the fraction shared-approach, so heuristics are used to speed-up the calculation. Based on the initial distance matrix, a single-linkage guide tree is constructed [6]. Sequence profiles are progressively aligned along the guide tree until all the input sequences are aligned.

A variation of the Needleman-Wunsch algorithm [7] is used for global profile alignment, as carried out by the "AlignProfiles" function in the DECIPHER software. First, anchors to reduce the dynamic programming matrix are automatically calculated based on ordered 7-mers for amino acid sequences. The two sequence sets being aligned must share a minimum fraction of exact matches (by default 70%) in order for a position to be used as an anchor. Likewise, the dynamic programming matrix is reduced in size by automatically restricting the set of possible tracebacks to paths that have reasonably high scores. This effectively avoids the corners of the dynamic programming matrix that are very unlikely to be used during the traceback step. Reducing the "alignment space" in this way is designed to make computation more time and memory efficient on long sequences without altering the results. In practice these heuristics work well unless the input sequences have substantially different lengths, in which case the user can adjust the "anchor" or "restrict" input parameters.

Filling the dynamic programming matrix is performed using a version of the profile sum-of-pairs (PSP) function [5]. The function has been modified to incorporate secondary structure probabilities from step 1:

$$PSP\_mod^{xz} = (1 - f_G^{\,x})(1 - f_G^{z})\left[\sum_i^{AA}\sum_j^{AA} f_i^{\,x} f_j^{\,z} S_{ij} + \sum_i^{HEC}\sum_j^{HEC} p_i^x p_j^z HEC_{ij}\right]$$

In the above equation, x and z are positions in the two sequence profiles being aligned, f_G is the fraction of gaps, f_i is the normalized fraction of amino acid i in the profile position, S_{ij} is the substitution matrix score for aligning amino acid i and j, p_i is the secondary structure probability of state i, and HEC_{ij} is the score for aligning two secondary structure states. By default, DECIPHER uses the MIQS substitution matrix [8] for S_{ij}. In determining f_G, f_i, and p_i, the sequences are weighted according to their divergence on the guide tree to account for uneven sampling [9].

The model of gap costs is based on the empirical observation that gap lengths are best approximated by a Zipfian distribution [10]. Therefore, the penalty for extending an open gap by another position is proportional to the length of the gap raised to a power (by default -1), as has been described previously [11]. Furthermore, gap opening and extension cost are varied linearly according to the divergence between the two sequence profiles being aligned [9]. In this way, the cost for a gap is more negative (costly) for closely related sequences than it is for divergent sequences. The baseline cost for opening a gap is adjusted according to the surrounding residues as specified by the model of gap placement:

$$GO_i = GO + \sum_{\substack{k=-4,\\ k\neq 0}}^{+4} \sum_j^{AA} f_{(i+k)j} G_{kj}$$

Where GO_i is the cost of opening a gap at position i, GO is the baseline gap opening penalty, $f_{(i+k)j}$ is the normalized fraction of amino acid j in position $i + k$, and G_{kj} is the log-odds (in third-bits) of observing amino acid j at position k (Table S1).

In step 3, the hamming distance between each pair of sequences is re-computed based on the alignment generated in step 2. A new UPGMA guide tree is constructed and the sequences are progressively realigned. In this step realignments are only necessary when the first and second trees differ below a node [5]. Iteration in step 3 results in a modest improvement over step 2, but this effect is subject to diminishing returns. Therefore, by default DECIPHER performs one iteration in step 3, although this can be adjusted by the user with the "iteration" parameter.

In step 4, the alignment is refined by splitting all of the aligned sequences into two sets at an edge of the guide tree and realigning both profiles [5]. This process results in two (oftentimes different) alignments, and the alignment corresponding to the highest sum-of-pairs score is kept. DECIPHER splits the original alignment at all edges of the UPGMA tree separating groups of sequences with greater than 70% (for amino acid sequences) average distance. This effectively realigns groups of sequences that are particularly difficult to align, and where the original alignment order may have been ineffective. By default the refinement process occurs once, although minor improvements may be observed by setting the "refinements" parameter to a number greater than 1 (the default).

FIGURES

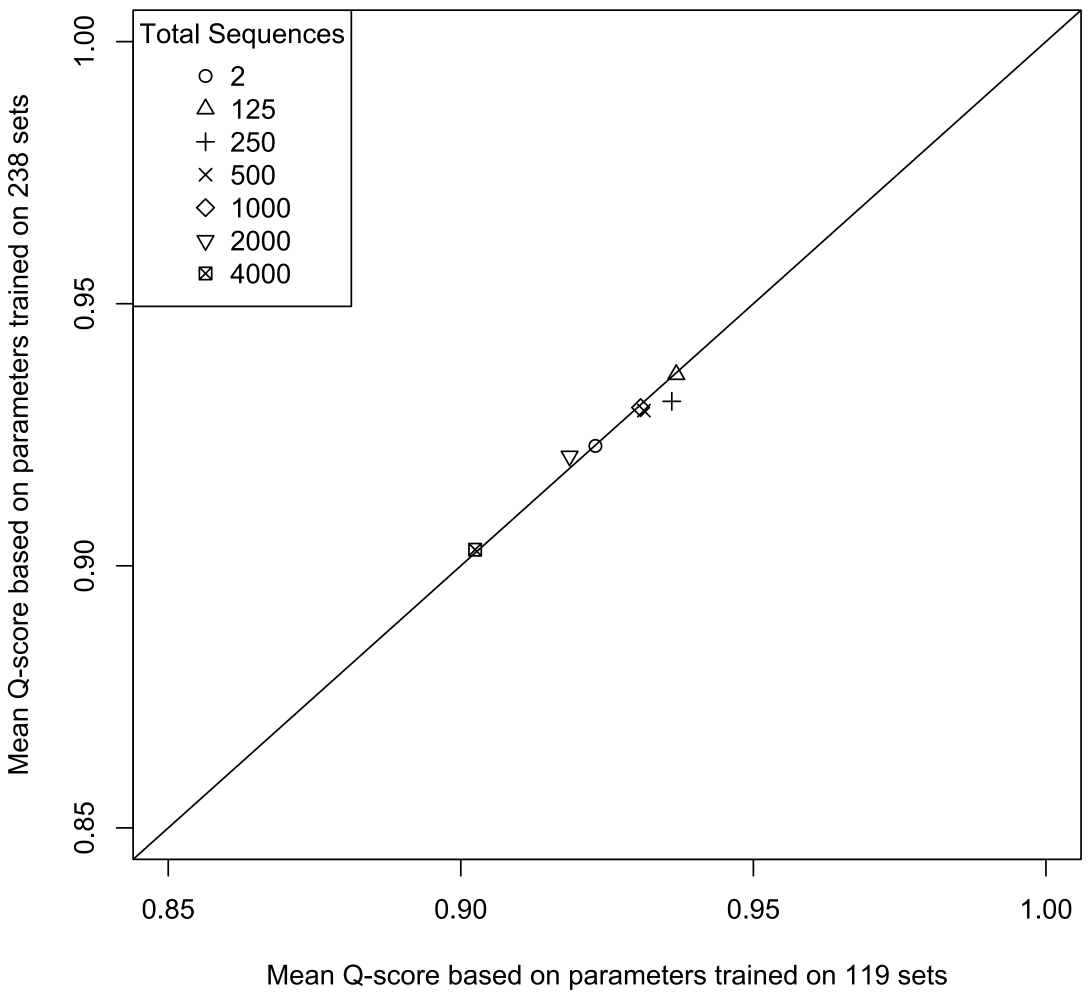

Figure S1. Secondary structure parameters are robust to the available training data. The six values in the secondary structure matrix were trained on 238 reference sets of HOMSTRAD-mod, or half of these sets (119). The mean Q-score was calculated for each set of parameters using the remaining 598 reference sets that were not used in parameter training. The results were nearly identical for both training sets, differing by a maximum of 0.4%. This result indicated that the six parameters in the secondary structure matrix were not over-trained on the HOMSTRAD-mod dataset.

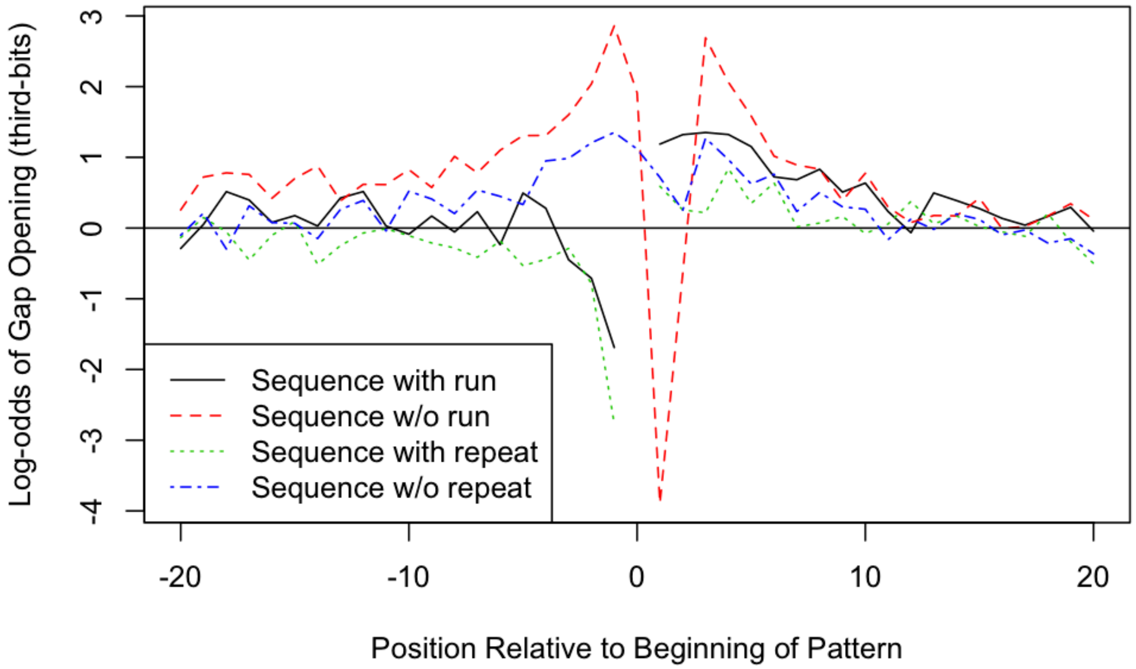

Figure S2. Likelihood of a gap opening at various positions around a pattern beginning at position zero. In pairwise alignments, gaps were substantially more likely to start before runs of three residues (e.g., AAA) or longer in the sequence without (w/o) the run. Gaps were much less likely to begin immediately after the start of a run in the opposing sequence (e.g., AAA/A--). Heteropeptide repeats of periodicity 2 (e.g., ACAC) to 6 showed a much smaller effect on the likelihood of a nearby gap opening. The discontinuity in the line for the sequences with the pattern exists because, by definition, a gap cannot be the start of a sequence pattern.

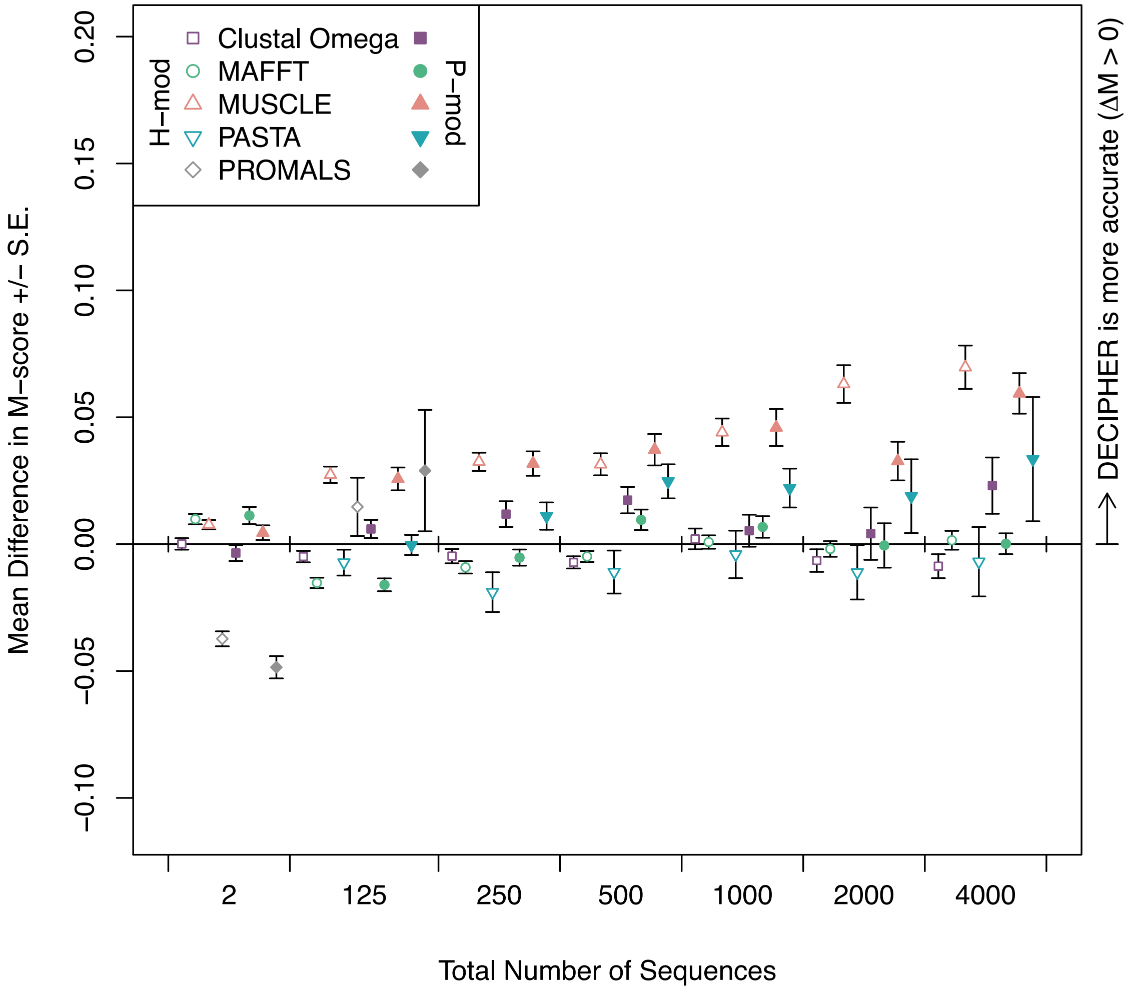

Figure S3. Modeler (M) score [12] relative to DECIPHER for Clustal Omega [13], MAFFT [14], MUSCLE [5], PASTA [15] and PROMALS [16] on HOMSTRAD-mod (H-mod) and PREFAB-mod (P-mod).

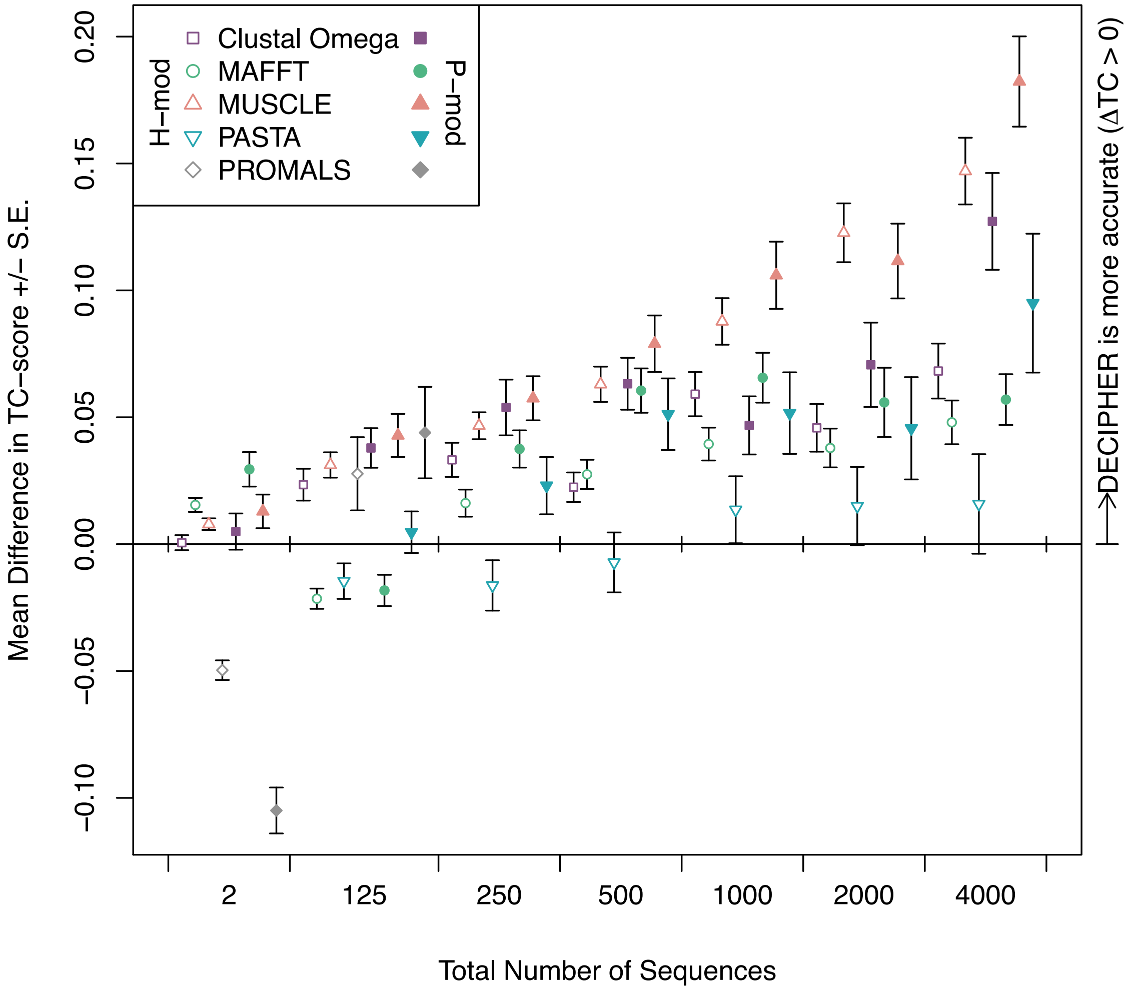

Figure S4. Total column (TC) score relative to DECIPHER for Clustal Omega [13], MAFFT [14], MUSCLE [5], PASTA [15], and PROMALS [16] on HOMSTRAD-mod (H-mod) and PREFAB-mod (P-mod).

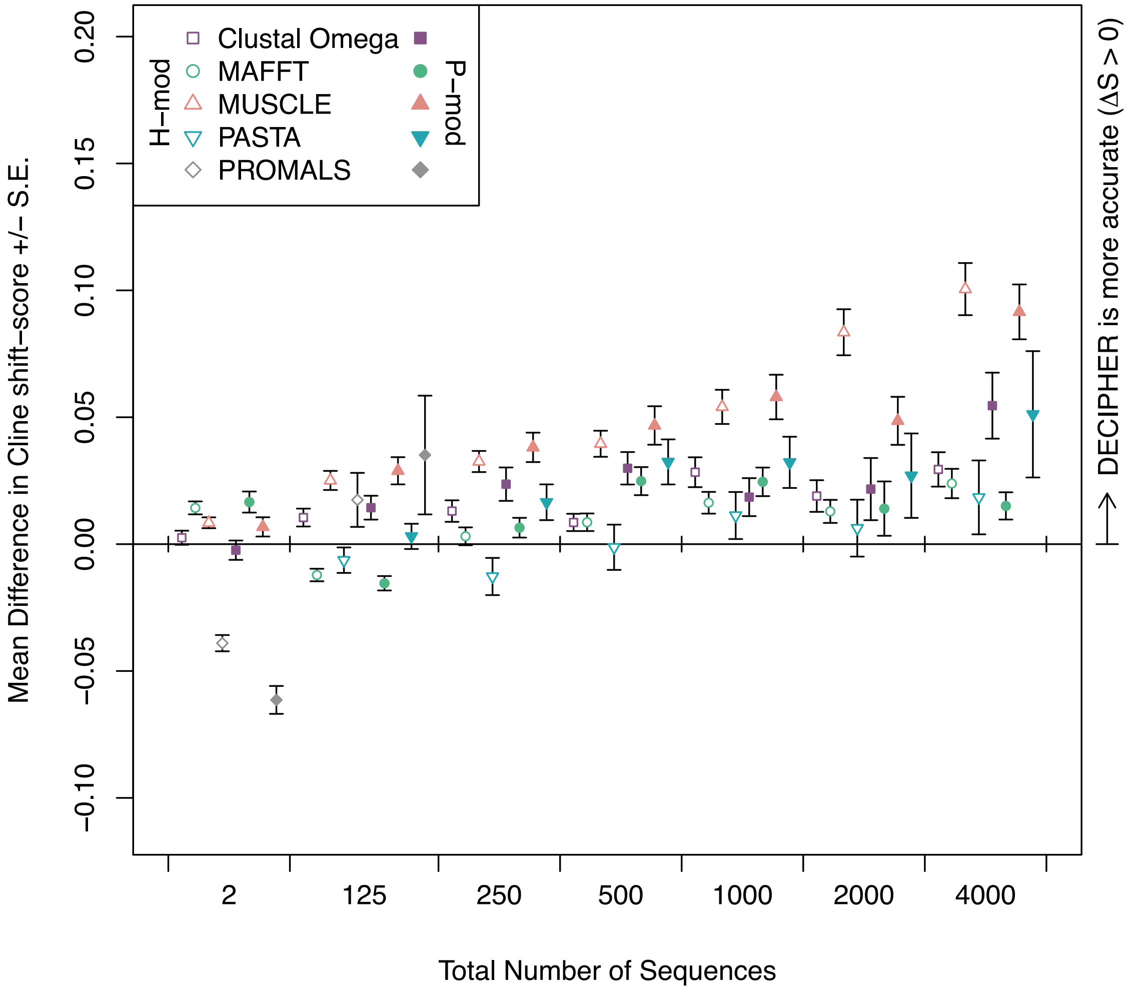

Figure S5. Cline shift-score (S) [17] relative to DECIPHER for Clustal Omega [13], MAFFT [14], MUSCLE [5], PASTA [15], and PROMALS [16] on HOMSTRAD-mod (H-mod) and PREFAB-mod (P-mod).

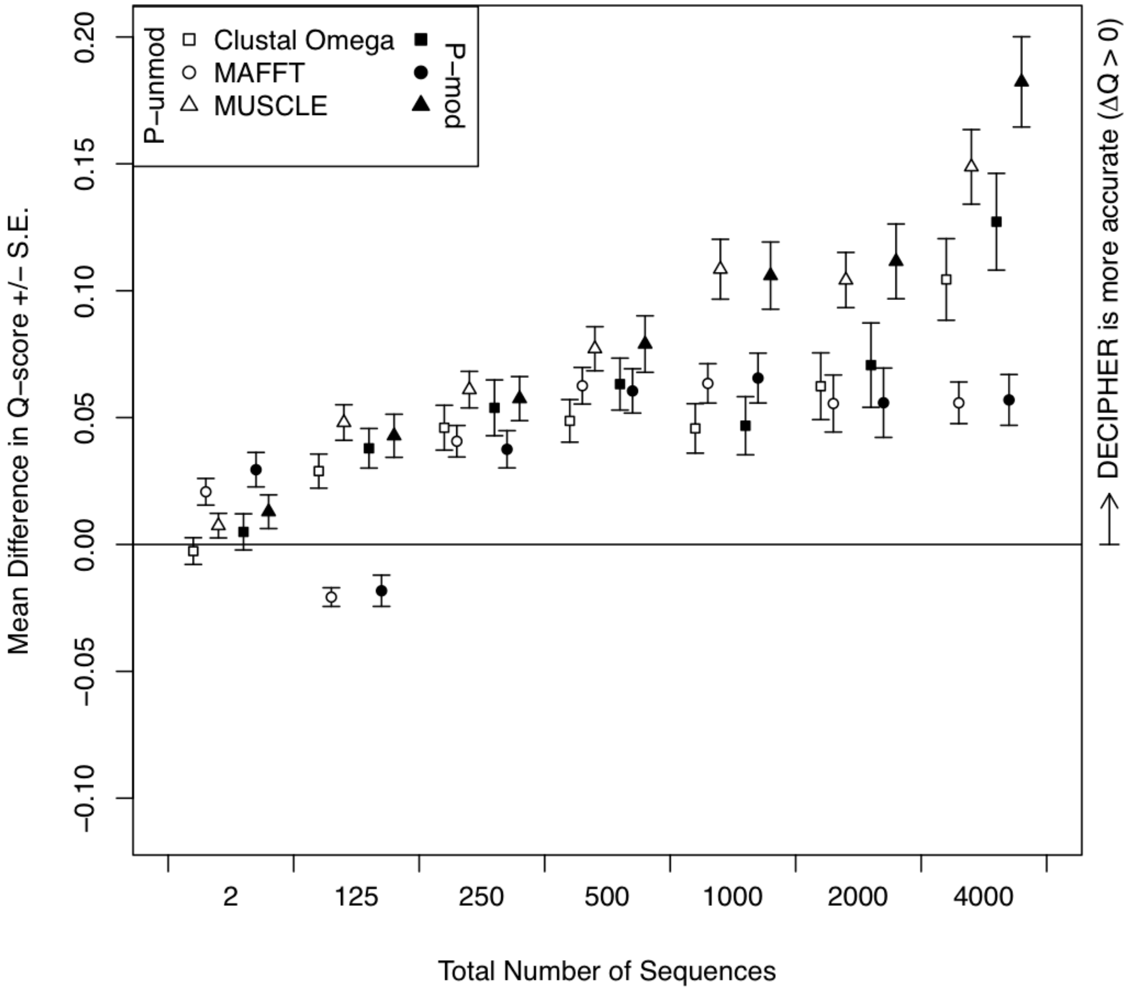

Figure S6. Q-score relative to DECIPHER for Clustal Omega [13], MAFFT [14], and MUSCLE [5] on the original (P-unmod) and modified (P-mod) PREFAB [18] reference sequences with an increasing number of total input sequences.

TABLES

Table S1. Log-odds (units of third-bits) of observing different amino acids near a gap in the One Gap Database [10].

AA/Position$^{\alpha}$	-4	-3	-2	-1	0	+1	+2	+3	+4
A	0.165	0.189	0.169	-0.569	-0.050	-0.511	-0.204	-0.190	-0.131
R	0.216	0.129	-0.104	0.146	-0.354	-0.045	-0.230	-0.331	-0.258
N	0.090	0.330	0.416	0.468	0.486	0.769	0.497	0.207	-0.035
D	-0.067	0.449	0.294	1.115	0.016	1.327	0.998	0.531	0.286
C	-0.173	-0.442	-0.748	-0.875	-0.014	-0.643	-0.864	-0.356	-0.070
Q	0.329	0.332	0.494	0.465	0.611	0.156	0.013	0.013	-0.152
E	0.372	0.690	0.774	0.803	0.037	0.772	0.490	0.409	0.428
G	-0.146	0.327	0.723	1.146	0.642	1.039	0.585	0.258	-0.020
H	-0.324	-0.218	-0.301	-0.415	0.107	-0.793	-0.184	-0.292	-0.285
I	-0.699	-1.179	-1.459	-1.754	-1.229	-1.270	-1.098	-0.456	-0.332
L	-0.112	-0.568	-0.868	-1.099	-0.788	-1.167	-1.140	-0.710	-0.535
K	0.571	0.667	0.856	0.998	0.178	0.617	0.540	0.398	0.183
M	-0.908	-1.143	-1.470	-1.508	-1.346	-2.049	-1.858	-1.591	-1.652
F	-0.453	-0.689	-1.041	-1.189	-0.718	-1.264	-1.207	-0.717	-0.350
P	0.554	0.473	0.960	1.124	1.030	0.621	1.287	1.148	0.782
S	0.432	0.533	0.629	0.480	1.265	0.637	0.783	0.459	0.486
T	-0.015	0.033	0.261	-0.005	0.441	-0.036	0.405	0.380	0.429
W	-0.515	-0.812	-1.285	-0.988	-0.916	-1.166	-1.507	-0.628	-0.292
Y	-0.378	-0.736	-1.090	-1.332	-1.127	-1.095	-1.216	-0.815	-0.455
V	-0.537	-0.870	-1.155	-1.233	-0.728	-0.462	-0.437	-0.171	0.136

$^{\alpha}$ Position relative to the gap in the pairwise alignment. Negative positions are to the left of the gap and positive positions are to the right of the gap in the sequence with the gap. Position zero corresponds to residues across from the gap in the sequence without the gap.

Table S2. The statistical significance (t-test p-values) of observed differences in Q-score between DECIPHER and other programs on two benchmark datasets.

	Total Sequences	Clustal Omega	MAFFT	MUSCLE	PASTA	PROMALS
HOMSTRAD-mod	2	0.424 (+)	1.69e-08 (+)	3.18e-04 (+)	N/A	< 1e-16 (-)
	125	3.09e-05 (+)	1.08e-07 (-)	1.51e-13 (+)	0.00108 (-)	0.00119 (+)
	250	2.67e-06 (+)	0.00666 (+)	< 1e-16 (+)	4.91e-04 (-)	N/A
	500	2.85e-05 (+)	8.01e-06 (+)	< 1e-16 (+)	0.295 (-)	N/A
	1000	1.84e-11 (+)	1.07e-08 (+)	< 1e-16 (+)	0.128 (+)	N/A
	2000	7.55e-07 (+)	2.58e-06 (+)	< 1e-16 (+)	0.337 (+)	N/A
	4000	8.79e-11 (+)	2.06e-08 (+)	< 1e-16 (+)	0.136 (+)	N/A
PREFAB-mod	2	0.244 (+)	8.98e-06 (+)	0.0262 (+)	N/A	< 1e-16 (-)
	125	8.39e-07 (+)	0.00163 (-)	3.49e-07 (+)	0.283 (+)	0.00231 (+)
	250	7.22e-07 (+)	2.55e-07 (+)	6.42e-11 (+)	0.0209 (+)	N/A
	500	9.04e-10 (+)	1.01e-11 (+)	3.81e-12 (+)	1.64e-04 (+)	N/A
	1000	2.81e-05 (+)	5.84e-11 (+)	1.3e-14 (+)	7.25e-04 (+)	N/A
	2000	1.27e-05 (+)	1.74e-05 (+)	2.6e-13 (+)	0.0114 (+)	N/A
	4000	1.23e-10 (+)	2.23e-08 (+)	< 1e-16 (+)	7.23e-06 (+)	N/A

DECIPHER's average Q-score was higher in cases with (+), lower with (-), and not compared when "N/A".

REFERENCES

1. Garnier J, Gibrat JF, Robson B: **GOR method for predicting protein secondary structure from amino acid sequence.** *Meth Enzymol* 1996, **266**:540–553.

2. Li Q, Dahl DB, Vannucci M, Hyun Joo, Tsai JW: **Bayesian Model of Protein Primary Sequence for Secondary Structure Prediction**. *PLoS ONE* 2014, **9**:e109832.

3. Kloczkowski A, Ting KL, Jernigan RL, Garnier J: **Combining the GOR V algorithm with evolutionary information for protein secondary structure prediction from amino acid sequence**. *Proteins* 2002, **49**:154–166.

4. Katoh K, Misawa K, Kuma K-I, Miyata T: **MAFFT: a novel method for rapid multiple sequence alignment based on fast Fourier transform.** *Nucleic Acids Research* 2002, **30**:3059–3066.

5. Edgar RC: **MUSCLE: a multiple sequence alignment method with reduced time and space complexity**. *BMC Bioinformatics* 2004, **5**:113.

6. Sievers F, Hughes GM, Higgins DG: **Systematic exploration of guide-tree topology effects for small protein alignments.** *BMC Bioinformatics* 2014, **15**:338.

7. Needleman SB, Wunsch CD: **A general method applicable to the search for similarities in the amino acid sequence of two proteins.** *J Mol Biol* 1970, **48**:443–453.

8. Yamada K, Tomii K: **Revisiting amino acid substitution matrices for identifying distantly related proteins.** *Bioinformatics* 2014, **30**:317–325.

9. Thompson JD, Higgins DG, Gibson TJ: **CLUSTAL W: improving the sensitivity of progressive multiple sequence alignment through sequence weighting, position-specific gap penalties and weight matrix choice.** *Nucleic Acids Research* 1994, **22**:4673–4680.

10. Chang MSS, Benner SA: **Empirical Analysis of Protein Insertions and Deletions Determining Parameters for the Correct Placement of Gaps in Protein Sequence Alignments**. *J Mol Biol* 2004, **341**:617–631.

11. Chenna R, Gibson T: **Evaluation of the suitability of a Zipfian gap model for pairwise sequence alignment**. 2011:1–6.

12. Sauder JM, Arthur JW, Dunbrack RL: **Large-scale comparison of protein sequence alignment algorithms with structure alignments.** *Proteins* 2000, **40**:6–22.

13. Sievers F, Wilm A, Dineen D, Gibson TJ, Karplus K, Li W, Lopez R, McWilliam H,

Remmert M, Soding J, Thompson JD, Higgins DG: **Fast, scalable generation of high-quality protein multiple sequence alignments using Clustal Omega**. *Molecular Systems Biology* 2011, **7**:1–6.

14. Katoh K, Standley DM: **MAFFT Multiple Sequence Alignment Software Version 7: Improvements in Performance and Usability**. *Molecular Biology and Evolution* 2013, **30**:772–780.

15. Mirarab S, Nguyen N, Guo S, Wang L-S, Kim J, Warnow T: **PASTA: Ultra-Large Multiple Sequence Alignment for Nucleotide and Amino-Acid Sequences.** *J Comput Biol* 2014.

16. Pei J, Grishin NV: **PROMALS: towards accurate multiple sequence alignments of distantly related proteins**. *Bioinformatics* 2007, **23**:802–808.

17. Cline M, Hughey R, Karplus K: **Predicting reliable regions in protein sequence alignments.** *Bioinformatics* 2002, **18**:306–314.

18. Edgar RC: **MUSCLE: multiple sequence alignment with high accuracy and high throughput**. *Nucleic Acids Research* 2004, **32**:1792–1797.
